# Protective Effect of Spirulina-Derived C-Phycocyanin against Ultraviolet B-Induced Damage in HaCaT Cells

**DOI:** 10.3390/medicina57030273

**Published:** 2021-03-16

**Authors:** Young Ah Jang, Bo Ae Kim

**Affiliations:** 1 Convergence Research Center for Smart Healthcare of KS R & DB Foundation, Kyungsung University, Busan 48434, Korea; yaviol@ks.ac.kr; 2 Department of Cosmetics Beauty, College of Technology Sciences, Mokwon University, Doanbuk-ro 88, Seo-gu, Daejeon 35349, Korea

**Keywords:** C-phycocyanin, UVB, matrix metalloproteinase, ROS, involucrin, filaggrin, loricrin

## Abstract

*Background and objectives:* Reactive oxygen species (ROS) overwhelm the antioxidant defense system, induce oxidative stress, and increase matrix metalloproteinase (MMP) expression, resulting in skin aging. Thus, preventing ultraviolet B (UVB)-induced skin damage can attenuate skin aging. Spirulina (a biomass of cyanobacteria, also called blue-green algae) is comprised of prokaryotes, whereas microalgae are eukaryotes and are rich in phycocyanin, a powerful antioxidant. *Materials and Methods:* Here, we investigated the photoprotective effects of spirulina-derived C-phycocyanin (C-PC) against UVB radiation using keratinocytes (HaCaT cells). *Results:* UVB radiation increased MMP-1 and MMP-9 expression but decreased involucrin, filaggrin, and loricrin expression. C-PC showed no toxicity at concentrations of 5–80 μg/mL in terms of HaCaT cell viability. UVB-irradiated HaCaT cells had a 50.8% survival rate, which increased to 80.3% with C-PC treatment. MMP expression increased with UVB treatment, whereas MMP-1 and MMP-9 concentrations decreased with C-PC treatment. UVB reduced involucrin, filaggrin, and loricrin expression in HaCaT cells, but 80 μg/mL C-PC increased their expression by >25%. In the UVB radiation group, dichlorofluorescin diacetate fluorescence intensity in HaCaT cells increased by 81.6% compared with that in the control group, whereas ROS production was reduced by 51.2% and 55.1% upon treatment with 40 and 80 μg/mL C-PC, respectively. *Conclusions:* C-PC might reduce or prevent skin aging by reducing UVB irradiation-induced skin wrinkles and free radicals.

## 1. Introduction

The skin is the largest organ in humans and is also the first line of defense against the outside environment. When a person is exposed to sunlight, ultraviolet (UV) rays act directly on the epidermis, the outermost part of the skin, which is composed of keratinocytes [1]. Skin aging can be attributed to the action of exogenous and endogenous factors, which cause wrinkles, sagging, and relaxation [2]. UV rays from sunlight, especially UVB, with a wavelength of 280–320 nm, cause significant changes in the skin tissue by destroying collagen, a major component of the extracellular matrix (ECM) [3]. In photoaging, UV exposure promotes the production of reactive oxygen species (ROS), including singlet oxygen, superoxide anion radical, hydrogen peroxide, hydroxyl radical, alkoxyl radical, and hydroperoxyl radical. This abnormal increase in ROS levels damages the body’s antioxidant defenses, induces oxidative stress, promotes inflammatory cytokine secretion in keratinocytes of the epidermis, and induces matrix metalloproteinases (MMPs) in dermal fibroblasts, thus promoting skin aging [4]. Even at extremely low levels of UVB irradiation, ROS are generated in the exposed human skin and initiate protein oxidation, DNA damage, and lipid peroxidation. ROS also promote the expression of MMPs, which accelerate skin aging by mediating the decomposition of collagen and elastin fibers, components of the ECM [5,6,7,8]. MMPs degrade collagen, and the decomposed degraded collagen is active in the human body and affects the regulation of the fibroblast phenotype during aging [9,10,11]. Collagen is known as a functional ingredient in cosmetics because it has excellent effects on skin moisturizing and elasticity. In addition, involucrin, filaggrin, and loricrin are major proteins that play important roles in formation of the epidermal skin barrier and in skin hydration [12,13]. UVB-induced inflammatory responses include increases in the levels of proinflammatory cytokines, such as tumor necrosis factor-α (TNF-α), interleukin-1β (IL-1β), and IL-6, all of which accelerate skin damage and activate MMPs [14]. There is considerable evidence that UV-induced oxidative stress mediates activation of protein kinases such as mitogen-activated protein kinases (MAPKs) in the body through a series of cascades. UVB-induced MAPK phosphorylation is involved in various skin diseases, such as skin cancer and photoaging [15,16,17,18]. To protect the body against these oxidative environments, the skin has an antioxidant defense system, consisting of antioxidant enzymes such as superoxide dismutase (SOD), catalase, and glutathione peroxidase and non-enzymatic antioxidants such as vitamins E and C and glutathione. However, excessive ROS, produced upon continuous UV exposure, can disrupt the skin’s antioxidant defenses, accelerating skin aging due to oxidative stress. Therefore, it is important to develop an antioxidant defense network to inhibit the production of excess free radicals in the skin or efficiently remove the generated free radicals and protect human skin cells from oxidative damage.

UV radiation (UVR) has a positive and a negative part that induces damage in the skin. According to the papers of existing researchers, excessive UVR is known to induce various skin diseases and aging by being involved in skin irritation and inflammatory reactions. However, phototherapy is used to treat skin inflammation, pigmentation, and various skin diseases. In addition, phototherapy affects the endocrine system, nerves, rheumatoid arthritis, inflammatory bowel disease, multiple sclerosis, body metabolism, food intake, and appetite control [19]. In this study, we focused on the damage of skin keratinocytes by UVR, and tried to verify the effectiveness of the natural product-derived compounds useful for skin cells.

**Figure 1:**
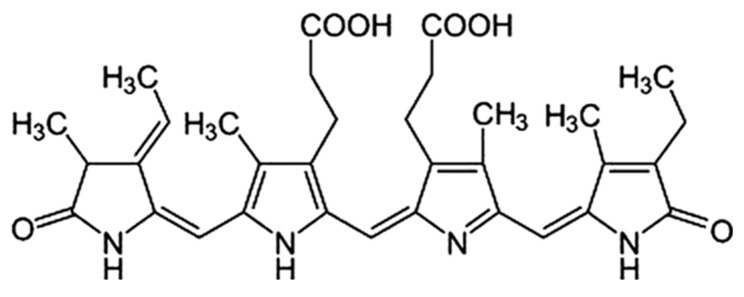
Chemical structure of C-phycocyanin.

Spirulina is a fine biomass of blue-green algae, or cyanobacteria, of the genus *Arthrospira*. Spirulina grows in alkaline and warm environments, including seawater and fresh water, in Asia, Africa, Europe, South America, and North America. In terms of nutrition, spirulina is a rich source of macronutrients, including high-quality proteins, iron, linolenic acid, vitamins, minerals, sulfide polysaccharides, and phycocyanin [20,21,22]. In food and nutrition, spirulina has been reported to be helpful in preventing or managing hypercholesterolemia, hyperglycerolemia, certain inflammatory conditions and allergies, and cardiovascular disease [23,24,25] In addition, the antioxidant activity in the preclinical stage was increased as a result of the ingestion of 0.18 g spirulina in rats for 7 weeks, and the enzyme activity increasing antioxidant activity was increased. This showed the effect of suppressing inflammation [26,27]. Spirulina is therefore of great interest because of its possible use as a functional food. Moreover, spirulina has been proven to have good water solubility for an organic ingredient that might be used as a food or nutritional supplement and is safe for human consumption because it does not exhibit acute or chronic toxicity [28,29]. The protein content of spirulina ranges from 60 to 70% of dry weight [30], which is an exceptional rate because the vast majority of plant-derived foods, even those known as good protein sources, contain approximately 35% protein. In fact, C-phycocyanin (C-PC), a molecule containing phycocyanobilin, a homolog of biliverdin, is one of the major proteins in spirulina and accounts for approximately 20% of dry algae [31,32]. Phycocyanin contains various bioactive compounds, which can potentially be applied as therapeutic or functional additives. C-PC, a bile protein found in blue-green algae such as spirulina, is used as an adjuvant due to its various advantageous activities including ROS inhibitory effects on the basis of various experimental results [33,34]. In addition, the anti-inflammatory activity of C-PC [35,36,37], including attenuation of lipopolysaccharide-induced iNOS expression and TNF production through the suppression of activation of nuclear factor in RAW 264.7 macrophages, as well as the inhibition of COX-2 activity, have been demonstrated in vitro [38]. However, the protective effects of phycocyanin against UVB-induced oxidative damage have not been sufficiently studied. In this study, we investigated whether phycocyanins could protect keratinocytes from UVB-induced damage. The chemical structure of C-PC is shown in Figure 1.

## 2. Material and Methods

### 2.1. Materials

C-PC from spirulina was purchased from Sigma-Aldrich (St. Louis, MO, USA). Dulbecco’s modified Eagle’s medium (DMEM), penicillin/streptomycin, and fetal bovine serum (FBS) were purchased from Gibco BRL (Grand Island, NY, USA). 2′,7′-Dichlorofluorescin diacetate (DCFDA) was purchased from Sigma-Aldrich (St. Louis, MO, USA). The EZ-Cytox cell viability assay kit was purchased from DOJEN Bio (DoGenBio Co., Ltd., Dogen, Seoul, Republic of Korea). Human total MMP-1 and MMP-9 enzyme-linked immunosorbent assay (ELISA) kits were obtained from R&D Systems (Minneapolis, MN, USA).

### 2.2. Cell Culture and UVB Irradiation

HaCaT cells (Korean Cell Line Bank, Seoul, Korea), a human dermal keratinocyte cell line, were cultured in DMEM containing 10% (*v*/*v*) FBS and antibiotics in a 37 °C, 5% CO_2_ incubator for 24 h. Cells were subcultured every 2–3 days. The UVB wavelength was 312 nm, and the damage to the cells was confirmed by irradiating the cells with UVB at 120 mJ/cm^2^. The sample and UVB irradiation treatment were as follows. Cells were treated for 24 h with various concentrations (5, 10, 20, 40, and 80 μg/mL) of C-PC and then exposed to UVB radiation at a dose of 40 mJ/cm^2^ (UV-X000; LAB24, Seoul, Korea) for 1 min. The final intensity of UVB irradiated on the top of plate surface was 0.6 mW/m^2^. Cells that did not receive C-PC pretreatment and were not exposed to UVB irradiation were used as a control.

### 2.3. Cytotoxicity Assay

HaCaT cells were seeded in 100 μL of the medium at a density of 1 × 10^4^ cells per well in a 96-well plate. After 24 h of incubation, the cells were treated with C-PC at the indicated concentrations and irradiated with UVB, followed by incubation for another 24 h. The EZ-Cytox cell viability assay reagent (10 μL per well) was added to the cell culture plate for 1 h, and then the absorbance was measured at 490 nm using a microplate reader (Bio-Rad Laboratories, CA, USA).

### 2.4. Measurement of MMP-1 and MMP-9 Secretion 

HaCaT cells were seeded in 96-well plates (5 × 10^4^ cells per well) and pretreated with C-PC using the same protocol. After exposure to UVB radiation, cell culture supernatants were centrifuged at 189× *g* for 5 min, and MMP-1 and MMP-9 levels were determined in the culture medium using the total MMP-1 (cat. no. DMP100) and MMP-9 (cat. no. DMP900) ELISA kits according to the manufacturer’s instructions. Levels of MMP-1 and MMP-9 were quantified using a fluorescence microplate reader (Molecular Devices, LLC, CA, USA).

### 2.5. Western Blotting

HaCaT cells were treated with C-PC in a 6-well plate (5 × 10^5^ cells per well) for 24 h and then washed twice with phosphate-buffered saline (PBS) and lysed in radioimmunoprecipitation (RIPA) buffer. The protein content was measured using a Bio-Rad protein assay kit (Hercules, CA, USA). The protein concentrations were quantified in the lysates, and 20 μL of protein was separated by electrophoresis on a 10% SDS-polyacrylamide gel. The separated proteins were transferred to a polyvinylidenefluoride (PVDF) membrane, and the membrane was blocked with 5% skim milk in tris-buffered saline tween TBST for 1 h at room temperature. Primary antibodies (Santa Cruz Biotechnology Inc., Santa Cruz, CA) against involucrin (sc-21748), filaggrin (sc-80609), and loricrin (sc-51103) were diluted to 1:1000 and incubated with the membranes overnight at 4 °C, followed by washing the membranes 3 times with TBST at 10 min intervals. The membranes were then incubated with the appropriate secondary antibody (sc-2004) diluted to 1000 for 2 h at room temperature. After washing the membranes three times, we detected immunoreactive bands, and their intensity was quantified using a LAS 4000 image analyzer (Fujifilm Life Sciences, Tokyo, Japan).

### 2.6. Measurement of ROS 

The compound DCFDA was used to measure the level of ROS production. HaCaT cells were treated as previously described and incubated with 10 μM DCFDA dye in a dish with a glass bottom for 20 min at 37 °C. Fluorescence intensity was observed under a fluorescence microscope (Nikon, Eclipse TS100 Epi-fluorescence, Tokyo, Japan) at an excitation wavelength of 488 nm and an emission wavelength of 525 nm.

### 2.7. Statistical Analysis

All measurements were conducted in triplicate, and the data are presented as the mean ± standard error of the mean. The results were analyzed using ANOVA, with Tukey’s multiple comparison test, and *p* < 0.05 was considered to indicate statistical significance.

## 3. Results

### 3.1. Protective Effect of C-PC against UVB-Induced Damage in HaCaT Cells

**Figure 2:**
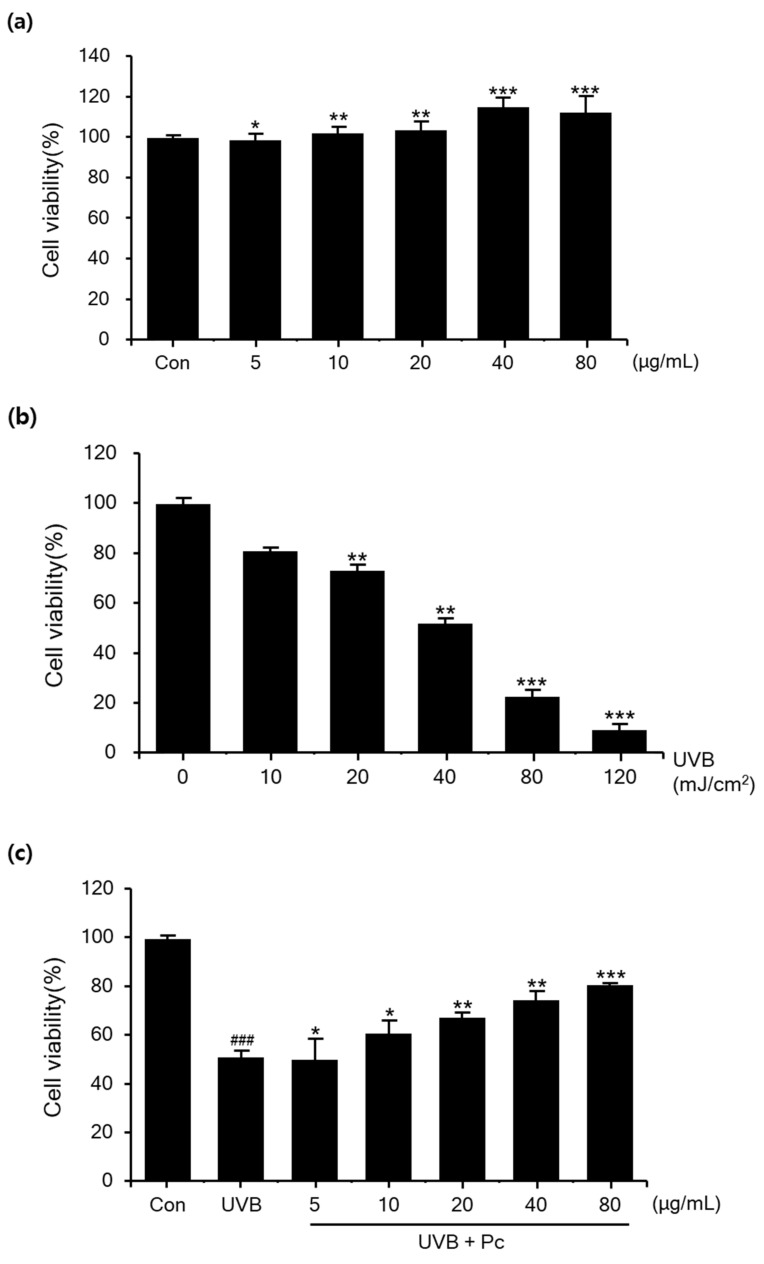
Cell viability of human keratinocytes after ultraviolet B (UVB) exposure. (**a**) HaCaT cell viability after pretreatment with various concentrations of C-phycocyanin (C-PC); (**b**) toxicity of UVB according to the dosage; (**c**) viability of cells treated with C-PC, followed by UVB irradiation. Data are presented as percentages of the control group. ### *p* < 0.001 vs. control group. * *p* < 0.05, ** *p* < 0.01, and *** *p* < 0.001 vs. UVB/vehicle group.

Before analyzing the protective effect of C-PC against UVB-induced damage in HaCaT cells, we tested the cytotoxicity of C-PC at each concentration (5, 10, 20, 40, and 80 μg/mL). The results showed that C-PC was not toxic at any concentration; instead, C-PC treatment at concentrations of 40 and 80 μg/mL increased the cell growth by 11.4% and 12.2%, respectively (Figure 2a). Next, cell viability after various doses of UVB was evaluated in HaCaT cells. We tested the cell-protective effects of PC on the basis of 40 mJ/cm^2^, which is a concentration that caused damage to cells (Figure 2b). When analyzing the protective effect of C-PC against UVB-induced damage in HaCaT cells, we found that UVB irradiation of untreated cells reduced their viability to 50.8% compared to that in the negative control group; however, when cells were pretreated with 80 μg/mL C-PC, their viability increased by 29.5% (Figure 2c). These data suggest that C-PC protects against UVB-induced cell damage.

### 3.2. Effect of C-PC on UVB-Induced Secretion of MMP-1 and MMP-9

**Figure 3:**
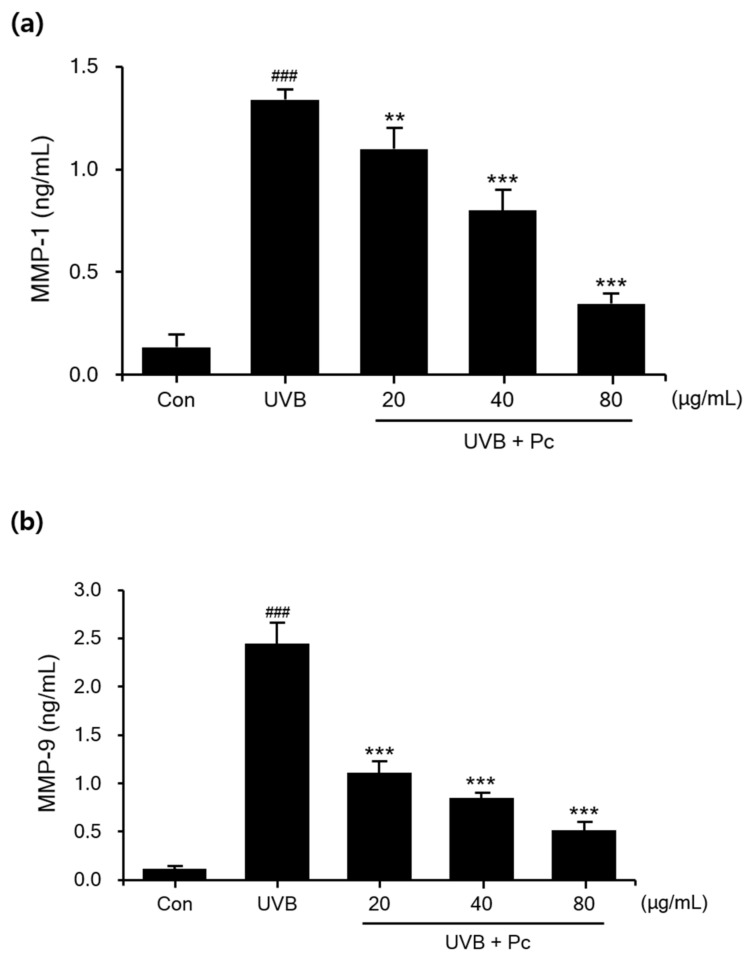
Effects of C-phycocyanin (C-PC) on matrix metalloproteinase (MMP)-1 and MMP-9 secretion by ultraviolet B (UVB)-induced HaCaT cells. Cells were treated with C-PC before UVB irradiation. The levels of secretion of (**a**) MMP-1 and (**b**) MMP-9 were measured in the culture medium of UVB-irradiated HaCaT cells. ### *p* < 0.001 vs. control group. ** *p* < 0.01 and *** *p* < 0.001 vs. UVB/vehicle group.

In a photoaged skin, dry skin and wrinkles are associated with increased expression of MMPs, such as MMP-1 and MMP-9, which break down the ECM. MMP-1 and MMP-9 expression experiments were performed using the Quantikine ELISA Kit, and the secretion of MMP-1 and MMP-9 was increased upon UVB irradiation of HaCaT cells. As the concentration of C-PC increased, the secretion of MMP-1 and MMP-9 was progressively inhibited compared with that in the UVB-irradiated group. The MMP-1 concentration was 10-fold higher and that of MMP-9 was 18-fold higher in the UVB-irradiated group compared to that than in the negative controls. In contrast, in the C-PC (80 μg/mL)-treated group, MMP-1 and MMP-9 levels were significantly reduced by 73.8% and 78.7%, respectively (Figure 3). Thus, the data suggest that C-PC inhibits the expression of MMPs and might suppress wrinkle formation due to aging.

### 3.3. Effect of C-PC on Involucrin, Filaggrin, and Loricrin Levels in UVB-Induced HaCaT Cells

**Figure 4:**
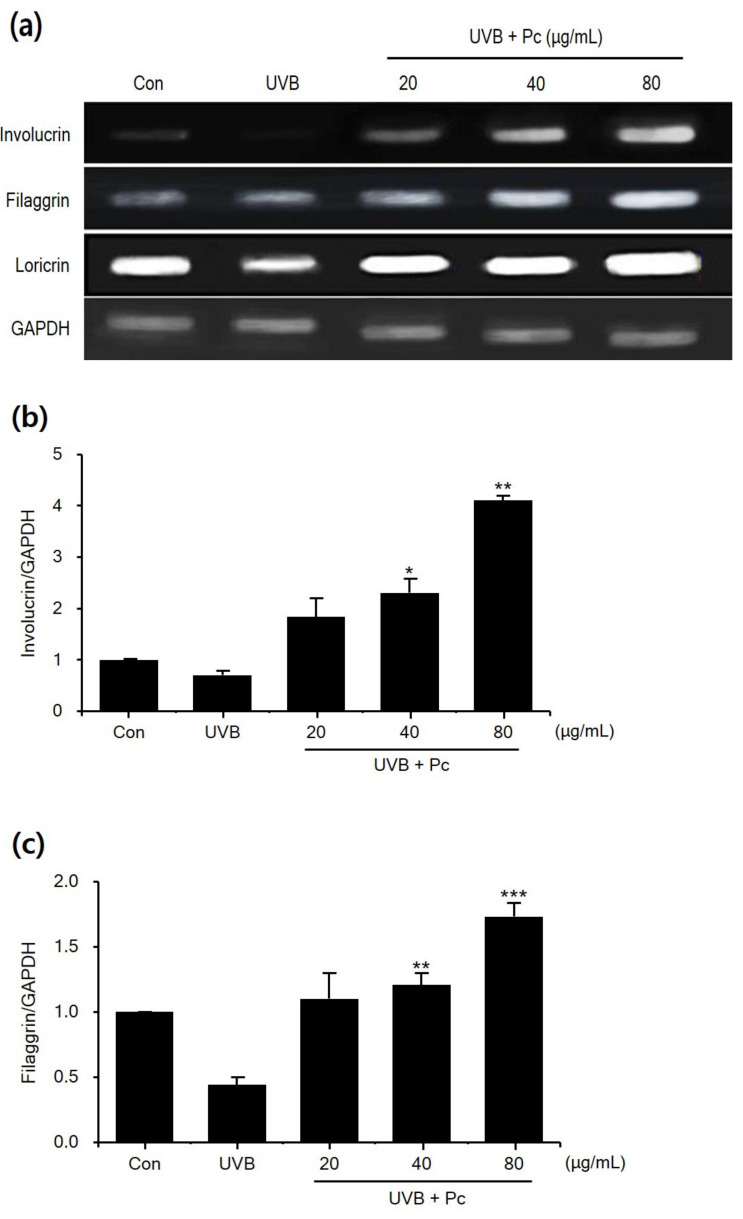

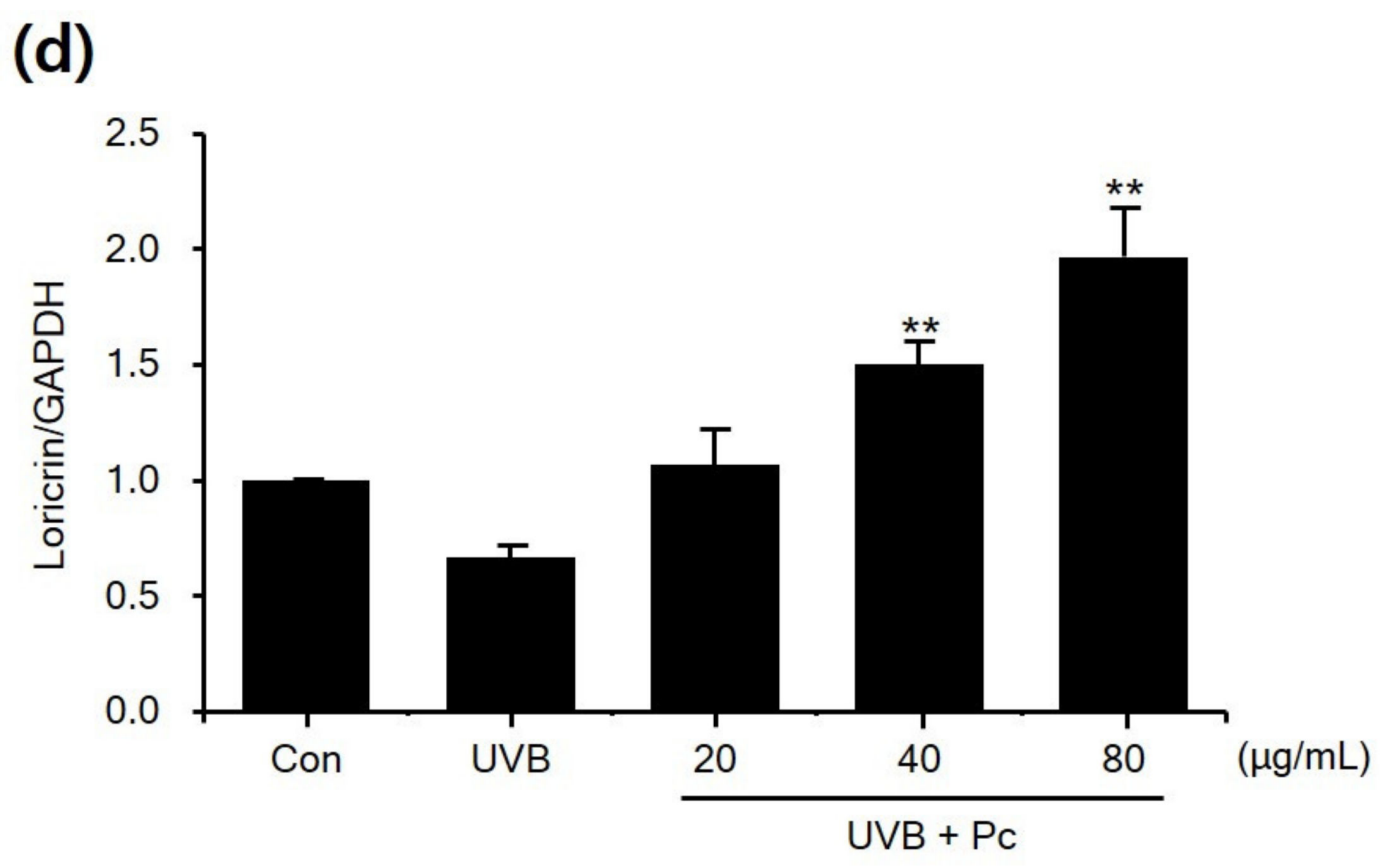
Effects C-phycocyanin (C-PC) on (**a**) western blot band (**b**) involucrin, (**c**) filaggrin, and (**d**) loricrin levels in ultraviolet B (UVB)-induced HaCaT cells, as assessed using Western blot analysis following treatment C-PC (20, 40, 80 μg/mL) These results are representative of three experiments performed in triplicate. Band intensities were quantified, and data are presented as the mean ± standard deviation. * *p* < 0.05, ** *p* < 0.01, and *** *p* < 0.005 vs. UVB/vehicle group.

To examine the effects of C-PC on regulators of skin barrier function, we studied the expression of skin barrier proteins (Figure 4). The result showed that the expression of involucrin, filaggrin, and loricrin was inhibited by UVB irradiation compared with that in the negative control group.

In the UVB group, the levels of involucrin, filaggrin, and loricrin were 30%, 66%, and 33% lower, respectively, than those in the negative control group. In contrast, in the C-PC (80 μg/mL)-treated group, the levels of involucrin, filaggrin, and loricrin were 5.8-, 3.9-, and 2.9-fold higher, respectively, than those in the UVB group. C-PC showed efficacy at all concentrations, and concentration-dependently increased the expression of the tested proteins. Therefore, these results indicate that C-PC might be able to restore the physical barrier function of the skin upon UVB damage.

### 3.4. Inhibition of ROS Production by C-PC in UVB-Induced HaCaT Cells

**Figure 5:**
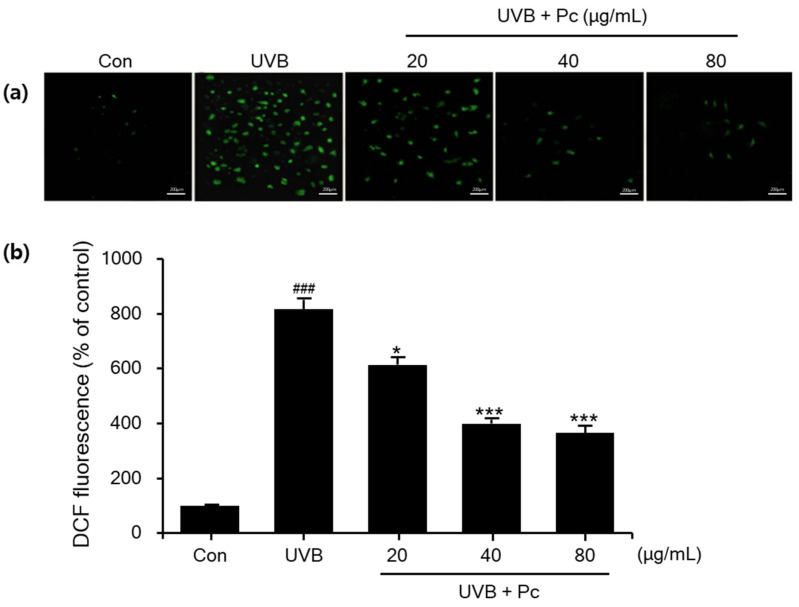
Effects of C-phycocyanin (C-PC) on ultraviolet B (UVB)-induced intracellular reactive oxygen species (ROS) levels. (**a**) HaCaT cells were seeded in 96-well plates and incubated with 2′,7′-dichlorofluorescin diacetate (DCFDA) dye prior to UVB exposure. (**b**) ROS levels were measured at 6 h post-UVB radiation along with treatment with the indicated concentrations of C-PC. Scale bars, 200 µm. Data are presented as the mean ± SD of three independent experiments. ### *p* < 0.001 vs. control group. * *p* < 0.01 and *** *p* < 0.001 vs. UVB/vehicle group.

Oxidative stress is an important event in both physiological and pathological conditions. In this study, we presented a method to quantify oxidative stress by measuring total ROS using 2′,7′-dichlorodihydrofluorescein diacetate (DCFDA) staining in colorectal cancer cell lines. DCFDA staining was used to analyze total ROS production in HaCaT cells and evaluate the intracellular antioxidant activity of C-PC using fluorescence microscopy image analysis. UVB treatment significantly increased the fluorescence intensity of DCFDA in HaCaT cells by 81.6% compared with that in the control group. Meanwhile, ROS production was reduced by 51.2% and 55.1% upon cell treatment with 40 and 80 μg/mL C-PC, respectively (Figure 5). Therefore, C-PC is effective in reducing UVB-induced ROS production in HaCaT cells, suppressing oxidative stress.

## 4. Discussion

UV rays reaching the earth’s surface are classified into UVB (280–320 nm) and UVA (320–400 nm), and their intensity and quantity vary greatly, depending on the region, season, and time of day. Excess ROS produced in the skin upon external stress, such as UV exposure, initiate lipid peroxidation in the cell membrane, damaging cells through a series of oxidation reactions. In the cell, hydrogen peroxide is formed via SOD-mediated catalysis, as well as in response to exposure to ionizing radiation such as UV rays [39]. In particular, the ionization of water in cells by ionizing radiation releases one electron from the water molecule and generates cationic water molecules (H_2_O+) and anionic water molecules (H_2_O-) [40]. Excess ROS damage cellular components such as proteins, DNA, and lipids and also promote the expression of MMPs, thereby accelerating skin aging through degradation of the tissue collagen matrix and elastin fibers. Therefore, antioxidants with intracellular ROS inhibition activity might attenuate skin aging. ROS exist in a balance with antioxidant factors in normal cells, but an excess of ROS or lack of antioxidant factors results in a state of increased intracellular oxidation power from the balanced state [41,42,43]. Exposure of the human skin to UVB radiation leads to the production of excessive ROS, which overpower the antioxidant defense system, causing oxidative stress [44]. ROS in biological systems are associated with skin cancer, DNA damage, skin aging, and many inflammatory disorders. UV light has been shown to cause a reduction in the levels of the antioxidant enzymes CAT and SOD [45]. In this study, C-PC was able to suppress the UVB-induced oxidative stress by regulating ROS to protect the antioxidant enzyme activity. MMPs are an extended family of degrading enzymes that make major contributions to tissue destruction in pathological processes, particularly arthritis, skin aging, and tumor invasion and metastasis [46]. It is known that MMPs break down collagen fibers forming a significant part of connective tissue in the skin and are involved in maintaining skin strength and elasticity [47,48,49,50]. The activity of MMPs, which is clearly related to substantial degradation of collagen fibers, has been shown to increase irradiation of human skin and even a single dose of UV light could affect the activity of MMPs. A reduction in procollagen expression due to repeated UV irradiation has been identified to be a source of photoaging [51]. This study found that treatment of HaCaT cells with C-PC prior to UVB irradiation significantly reduced MMP-1 and MMP-9 expression. Skin barrier function such as corneocyte, cornified envelope, and lamellar membrane appears through the stratum corneum structure composed of lipid, intercorneocyte lipid, and corneodesmosome [52]. Genes that increase expression related to skin differentiation include transglutaminase 1 and 3, involucrin, cornifin, loricrin, and filaggrin. When the differentiation of keratinocytes begins, transglutaminase transfers structural proteins such as involucrin, cornifin, and loricrin inside the cell membrane [53]. Combined, the stratum corneum forms the cornified cell envelope (CE), a rigid structure that plays the most important role in performing the skin barrier function. Early in the process of CE production, involucrin attaches to the cytoplasmic membrane. In the granule layer of keratinohyaline granule, loricrin is secreted and deposited on the desmosome of the cell membrane. In cells, filaggrin aggregates keratin to form macrofibrils [54]. The degraded filaggrin moves to the stratum corneum, forms the skin barrier, and is decomposed into free amino acids such as pyrrocarboxylic acid and trans-urocanic acid by the action of enzymes such as peptiylarginine-deiminase 3 (PAD3) [55]. The degradation of filaggrin into hygroscopic free amino acids is the main cause of natural moisturizing factors (NMF) produced in the stratum corneum. NMF is responsible for maintaining skin moisture and maintains moisture in the stratum corneum under low environmental humidity conditions [56]. In addition, trans-urocanic acid is converted to cis-urocanic acid (cis-UCA) by UVB radiation, which has been studied as a key mediator of UVB-induced inhibition [57].

In previous studies, the downregulation of filaggrin and loricrin was observed in in vitro re-constituted skin after UVB exposure [58,59]. These results showed that C-PC exerted a skin protection effect. Early protection is important because keratinocytes respond through major changes in inflammation and immune regulation observed after UVB exposure. Comprehensive results of the experiment showed that C-PC protected the skin from UV rays, inhibited MMP enzyme activity, and finally suppressed wrinkle formation. In addition, it had the effect of protecting filaggrin, involucrin, and loricrin, which are key factors in skin barrier relief. Therefore, it can be seen that C-PC has high potential as a cosmetic beauty material to protect against photoaging in the future.

## 5. Conclusions

C-PC exerts antioxidative effects against UVB-induced oxidative stress, which are mediated via the downregulation of MMPs and of ROS production, as well as by maintaining the expression of involucrin, filaggrin, and loricrin.
